# The impact of cardiovascular co-morbidities and duration of diabetes on the association between microvascular function and glycaemic control

**DOI:** 10.1186/s12933-017-0594-7

**Published:** 2017-09-15

**Authors:** F. Casanova, D. D. Adingupu, F. Adams, K. M. Gooding, H. C. Looker, K. Aizawa, F. Dove, S. Elyas, J. J. F. Belch, P. E. Gates, R. C. Littleford, M. Gilchrist, H. M. Colhoun, A. C. Shore, F. Khan, W. D. Strain

**Affiliations:** 1 Diabetes and Vascular Medicine Research Centre, Institute of Biomedical and Clinical Science and NIHR Exeter Clinical Research Facility, University of Exeter Medical School, Barrack Rd, Exeter, EX2 5AX UK; 2 Vascular and Inflammatory Diseases Research Unit, Division of Molecular and Clinical Medicine, School of Medicine, Ninewells Hospital and Medical School, University of Dundee, Dundee, DD1 9SY UK

**Keywords:** Diabetes, Cardiovascular disease, Microcirculation, Glycaemic legacy

## Abstract

**Background:**

Good glycaemic control in type 2 diabetes (T2DM) protects the microcirculation. Current guidelines suggest glycaemic targets be relaxed in advanced diabetes. We explored whether disease duration or pre-existing macrovascular complications attenuated the association between hyperglycaemia and microvascular function.

**Methods:**

743 participants with T2DM (n = 222), cardiovascular disease (CVD = 183), both (n = 177) or neither (controls = 161) from two centres in the UK, underwent standard clinical measures and endothelial dependent (ACh) and independent (SNP) microvascular function assessment using laser Doppler imaging.

**Results:**

People with T2DM and CVD had attenuated ACh and SNP responses compared to controls. This was additive in those with both (ANOVA p < 0.001). In regression models, cardiovascular risk factors accounted for attenuated ACh and SNP responses in CVD, whereas HbA_1_c accounted for the effects of T2DM. HbA_1_c was associated with ACh and SNP response after adjustment for cardiovascular risk factors (adjusted standardised beta (β) −0.096, p = <0.008 and −0.135, p < 0.001, respectively). Pre-existing CVD did not modify this association (β −0.099; p = 0.006 and −0.138; p < 0.001, respectively). Duration of diabetes accounted for the association between HbA_1_c and ACh (β −0.043; p = 0.3), but not between HbA_1_c and SNP (β −0.105; p = 0.02).

**Conclusions:**

In those with T2DM and CVD, good glycaemic control is still associated with better microvascular function, whereas in those with prolonged disease this association is lost. This suggests duration of diabetes may be a better surrogate for “advanced disease” than concomitant CVD, although this requires prospective validation.

**Electronic supplementary material:**

The online version of this article (doi:10.1186/s12933-017-0594-7) contains supplementary material, which is available to authorized users.

## Introduction

Whereas cardiovascular disease (CVD) is responsible for the majority of mortality in people with type 2 diabetes mellitus (T2DM), the microvascular complications, such as retinopathy and nephropathy, have the greatest impact on patient’s quality of life [1] and the economic cost of patients’ management [2]. Good glycaemic control has been demonstrated to halt, or at least slow, progression of these microvascular complications in *early* diabetes (within 12 months of diagnosis) in otherwise healthy people with diabetes [3, 4]. Recent trials, however, have suggested that this benefit is attenuated, or even absent in those with long-established complex T2DM (>5 years) [5–7]. Indeed, the ACCORD trial failed to show any benefits on microvascular complications from intensive glycaemic control and a paradoxical 22% increase in cardiovascular events, thought to be due to the adverse metabolic impact of hypoglycaemia and weight gain on a background of prolonged adverse glycaemic legacy. As a result, national and international guidelines now advocate relaxation of targets for glycaemic control in “advanced T2DM” [8] in order to reduce the potential side effects of therapy in a setting where the therapeutic potential of glycaemic control is limited. The term “advanced T2DM”, however, is ambiguous and open to interpretation. It is often defined interchangeably as a prolonged duration of diabetes or by the presence of complications such as CVD or renal disease. As most clinical trials have recruited patients based on a composite of these characteristics, it is difficult to determine which should play the dominant role in defining targets for glycaemic control. Recently, cardiovascular outcome trials such as LEADER [9] and Sustain-6 [10] have demonstrated a differential benefit on major adverse cardiac events (MACE) in those with pre-existing CVD compared to those at high risk of a cardiovascular event. In these studies, participants who were recruited without experiencing a previous event failed to gain any additional benefit from the GLP-1 based therapy, whereas treatment incurred a risk reduction in those who had experienced previous CV events. This raises doubts over the equal weight placed on pre-existing CVD/co-morbidities and duration of disease when it comes to target setting for glycaemic control.

When setting targets for glycaemic control in people with T2DM, physicians are often faced with individuals with new onset T2DM but pre-existing CVD, or conversely a long duration of T2DM in the absence of any macrovascular complications. These patients would not have been adequately considered in previous trials; in UKPDS, for example, only 7.5% of patients had pre-existing cardiovascular disease [3]. Given the principle role of good glycaemic control is to reduce progression of microvascular complications, physicians are left with the conundrum of how much weight to give each of these “target modifiers” (namely duration of T2DM and pre-existing CVD/co-morbidities) in establishing their therapeutic target.

In the absence of specific interventional studies, therefore, we rely on cross-sectional, “association” studies to inform and guide strategies for individualisation of treatment goals. Only a very small number of studies have attempted to address the effects of CVD risk, comorbidities and duration of T2DM on the association between glycaemic control and microvascular function. These have provided conflicting results, possibly due to the small number of patients involved in these studies [11–14]. As such it is unclear whether there is a disjunction between glycaemic control and progression of microvascular disease in advanced T2DM and whether this is a function of concomitant vascular disease in larger conduit vessels, shared risk factors with atherosclerotic disease, or simply pre-existing prolonged exposure to hyperglycaemia.

Structural changes in retinal vessel are associated with increased risk of CVD with some studies suggesting this might be true in women but not in men [15]. In diabetes retinal changes are believed to reflect systemic changes and have been suggested as a potential biomarker for microvascular complications [16]. These studies tend to address the relationship between structural changes within the microcirculation and DM and/or CVD with little information about functional changes to the microvessels.

Skin microcirculation is an established model to investigate systemic micro-vessel function [17, 18], further it has been independently associated with symptomatic coronary artery disease [19] and can be specifically interrogated to measure endothelium-dependent and -independent responses [20], thus providing information on *subclinical changes in vessel function*. In the present study, we explored the association between glycaemic control and microvascular function in patients with and without established T2DM and CVD.

We hypothesised that the diminished microvascular function previously described in those with T2DM will be a function of glycaemic control, whereas the impaired microvascular function in those with CVD will be a function of their conventional CVD risk factors. Further, we aimed to explore whether duration of T2DM, as a measure of adverse glycaemic legacy, or the presence of pre-existing CVD/co-morbidities, attenuated the association between glycaemic control and microvascular function.

## Methods

### Ethics approval

This study conformed to the *Declaration of Helsinki* and was approved by the National Research Ethics Service Southwest (09/H0202/49 and 10/H0206/67) and the East of Scotland Research Ethics Service (Tayside Committee; 10/S1402/44). All participants gave informed written consent.

### Study population

Participants were part of the “surrogate markers for micro- and macro-vascular hard endpoints for innovative diabetes tools” (SUMMIT) program (IMI Grant Number 115006; http://www.imi-summit.eu) and were recruited from EXTEND (Exeter 10000, NIHR Exeter Clinical Research Facility), the Royal Devon and Exeter NHS clinical service, the UK type 2 diabetes case–control collection Wellcome study and via the Scottish primary care research network, the Scottish diabetes research network research network, secondary care diabetes clinics and through advertising such as posters and leaflets. Participants were adult men and women, enhanced with individuals with T2DM and/or proven CVD. T2DM was diagnosed by the patients’ physician after two consecutive HbA_1_c above 48 mmol/mol. CVD was defined as previously described [21] and included a medical history of myocardial infarction, percutaneous coronary intervention (PCI), coronary arterial bypass graft (CABG), unstable angina and specialist diagnosed cerebrovascular event from Stroke and Cardiology units and clinics at the Royal Devon and Exeter Hospital and Ninewells Hospital, Dundee. CKD was defined as an estimated glomerular filtration rate of <60mL/min (estimated using the MDRD formula).

### Screening

Screening assessment included a medical history interview, including pharmacotherapy, electrocardiogram and anthropometry measures. Measures to a standard protocol included height, weight and waist-to-hip ratio [22]. Blood pressure was measured as the mean of three supine brachial measurements using an automated blood pressure device (Omron M6, Omron Healthcare Europe B.V. Hoofddorp, The Netherlands). Ankle-brachial index was calculated from the highest value of the ankle reading for posterior tibia or dorsalis pedis pressure, and the highest reading from the brachial artery (right or left). Measurements were taken using a Doppler probe (Doppler D900, Huntleigh Health Care, Cardiff, UK) and sphygmomanometer.

Twelve-hour-fasting blood samples were collected from all participants for the measurement of glucose and lipid concentrations either on the day of the study or within a week of the study day when not possible (Exeter and Dundee Pathology Services, Royal Devon and Exeter NHS Foundation Trust, Nantwell NHS trust), in accordance with the UK national quality assessment scheme.

### Microvascular assessment

All participants attended the clinical research facilities having refrained from food or drink (except water) at least 2 h before the visit, and avoided smoking, drinking tea or coffee, alcohol and strenuous exercise on the study day. Medications were omitted on the morning of the study, wherever possible. All studies were performed in temperature-controlled laboratories (23 ± 1 °C), with the participants lying in the supine position following an acclimatisation period of at least 20 min. The volar aspect of the right forearm was wiped gently with alcohol (Klercide 70/30) and then sterile water. Perspex direct electrode chambers [30 mm total diameter × 3 mm height, inner (drug) chamber 10 mm diameter] were attached using a double-sided adhesive ring, avoiding visible veins, freckles and hair. Chambers were filled with solutions under test; ACh (1% Miochol-E dissolved in mannitol, Novartis, Camberly, UK) and SNP (0.25%) (25 mg/mL Nitropress dissolved in 0.45% saline, Hospira, Lake Forest, USA). A glass cover slip was placed on the drug chamber to prevent reflection artefacts from the solution in the chamber when recording with the laser Doppler perfusion imager. An indifferent electrode was attached to the volar aspect of the participant’s wrist and connected to the chamber terminal to complete the circuit.

Identical solid-state laser Doppler imagers (LDI, Moor Instruments MODEL LDI2) were used in each centre (one in Exeter, one in Dundee) to measure skin microvascular perfusion, with the head of the LDI positioned 50 cm above the centre of the Perspex chamber. Skin microvascular perfusion is measured from the laser Doppler flux which is proportional to the average speed of red blood cells and their concentration. The LDI was set to scan a region of 4.8 cm^2^. The LDI was interfaced with a computer equipped with moorLDI windows PC dedicated software (research version 5.3). A battery-powered iontophoresis controller (MIC 1; Moor Instruments, Axminster, UK) was used to provide the current for iontophoresis.

ACh was delivered using five pulses (100 μA each) of anodal current with a 60 s interval between each dose (total charge 12 mC). Twenty-three consecutive 20 s recordings were made of forearm skin red blood cell flux (Additional file 1: Figure S1). There was no interval between these recordings.

SNP was delivered by cathodal current. A single pulse of 200 micro amps was introduced for 60 s (total charge 10 mC). Forearm skin red blood cell flux was again recorded in 20 s units, 21 times with no interval between scans.

Median perfusion response was calculated for each image obtained by the LDI using the total area to which the drug was applied. Skin microvascular perfusion was expressed in arbitrary units (arb.) and the peak response was measured and defined as the highest skin perfusion response reached during iontophoresis delivery of ACh and SNP.

### Endo-PAT: endothelial function

In a subgroup of 458 participants from the Exeter cohort (controls = 107, T2DM no CVD = 117, T2DM with CVD = 105, CVD only = 129) we assessed arterial endothelial function using Endo-PAT 2000 (Itamar Medical, Israel) measuring changes in the digital pulse waveform (PAT, peripheral Arterial Tone) by plethysmography following a 5 min occlusion. The baseline data were collected for a period of 10 min, a supra-systolic blood pressure cuff was then inflated on the upper arm of the non-dominant arm for exactly 5 min before instantaneous release and recording for a further 10 min.

The relative hyperaemia index (RHI) which reflects endothelial function is automatically calculated according to the following formula by the Endo-PAT2000 software.

$$ {\text{RHI}} = {{\left( {{{\text{A}} \mathord{\left/ {\vphantom {{\text{A}} {\text{B}}}} \right. \kern-0pt} {\text{B}}}} \right)} \mathord{\left/ {\vphantom {{\left( {{{\text{A}} \mathord{\left/ {\vphantom {{\text{A}} {\text{B}}}} \right. \kern-0pt} {\text{B}}}} \right)} {\left( {{{\text{C}} \mathord{\left/ {\vphantom {{\text{C}} {\text{D}}}} \right. \kern-0pt} {\text{D}}}} \right)}}} \right. \kern-0pt} {\left( {{{\text{C}} \mathord{\left/ {\vphantom {{\text{C}} {\text{D}}}} \right. \kern-0pt} {\text{D}}}} \right)}} \times {\text{Baseline correction }}\left( {\text{BC}} \right){\text{ factor}} $$

where, A is the mean amplitude of oscillation on test arm from 1.5 to 2.5 min post occlusion; B is the mean amplitude of oscillation on test arm during baseline; C is the mean amplitude of oscillation on control arm from 1.5 to 2.5 min post occlusion; D is the mean amplitude of oscillation on control arm during baseline.

### Statistical analysis

Where possible analysis was performed on continuous data to maximise power. Skewed variables were appropriately transformed, and geometric means (inter-quartile range are presented). Statistical significance for categorical variables was calculated using the Chi squared test and the ANOVA for continuous variables, with individual group differences assessed by Student’s t test. Analysis of co-variance was employed to determine explanations for differences between those with and without T2DM and CVD in key variables, with tests for interaction.

A result was deemed significant if p ≤ 0.05 for the ANOVA. In cases were comparisons are performed between four groups (i.e. six comparisons), a p value of <0.008 is regarded as significant. In the assessment of the optimal model which accounts for association between glycaemia and microvascular disease, where three models are compared, a p value of <0.017 is regarded as significant. Where multiple comparisons have occurred p values are reported in full for information purposes only. All analysis were performed using STATA (version 14.1, StataCorp, Texas USA).

In attempting to explain differences encountered between those with and without diabetes and/or CVD, multiple regression modelling was performed taking into account putative confounders or modifiers of the relationship. Confounding factors considered were age and sex, and mechanistic factors considered were obesity (weight, BMI, waist circumference, hip circumference and waist: hip ratio), blood pressure indices (brachial systolic, ankle systolic, brachial diastolic, mean arterial pressure, 24 h mean ambulatory blood pressure, day-time mean systolic blood pressure, night-time mean systolic blood pressure and day: night systolic blood pressure ratio) lipid profile (total cholesterol, HDL cholesterol, cholesterol: HDL ratio and fasting triglyceride) and smoking status. When considering the contribution of glycaemia, fasting glucose and HbA_1_c were considered. A single variable from each of these groups (body habitus, blood pressure, lipids, smoking and glycaemia) was included based on the greatest increase in the amount of variance explained by the model (R-squared value). The impact of adding additional variables in accounting for the variance of the model was tested by the likelihood ratio statistic (LRS). Polypharmacy (either as drug class or total tablet burden) were considered, however did not contribute to the model.

## Results

### Clinical characteristics

Those with T2DM had a higher HbA_1_c and BMI. Those with T2DM or CVD were more likely to be treated with anti-hypertensive and statins compared with the healthy controls (Table 1).

**Table 1:** Baseline characteristics stratified by group [mean (±SD) except were skewed data in which case geometric mean (IQ range) presented]

	Controls N = 161	CVD only = 183	DM no CVD = 222	DM with CVD = 177	p
Age (years)	64 [58–69]	68 [63–74]^a^	65 [59–71]^b^	69 [64–47]^a, c^	<0.001
Gender (M/F)	81/80	136/47	130/92	133/44	<0.001
Height (m)	1.69 (±0.1)	1.69 (±0.1)	1.69 (±0.1)	1.70 (±0.1)	0.428
Weight (kg)	76.0 (±13.7)	80.0 (±12.7)	91.7 (±16.5)^a, b^	90.2 (±16.6)^a, b^	<0.001
BMI (kg m^−2^)	26.59 (±4.19)	27.89 (±3.84)	31.93 (±5.59)^a, b^	30.98 (±4.93)^a, b^	<0.001
Systolic BP (mmHg)	137.5 (±18.2)	136.5 (±17.7)	137.2 (±14.9)	135.4 (±15.4)	0.644
Diastolic BP (mmHg)	79.2 (±8.9)	76.1 (±8.8)^a^	78.2 (±7.8)	74.1 (±8.6)^a, c^	<0.001
MAP (mmHg)	98.7 (±11.0)	96.2 (±10.6)	97.8 (±8.9)	94.5 (±9.2)^a, c^	<0.001
On anti-hypertensive (%)	50.3	86.3	79.7	94.3	<0.001
ABPI right leg	1.15 (±0.16)	1.11 (±0.19)	1.16 (±0.16)	1.12 (±0.26)	0.018
ABPI Left leg	1.13 (±0.14)	1.11 (±0.16)	1.15 (±0.16)	1.10 (±0.25)	0.042
Total cholesterol (mmol L^−1^)	5.4 [4.8–6.01]	4 [3.5–4.7]^a^	4.08 [3.5–4.64]^a^	3.7 [3.26–4.1]^a, b, c^	<0.001
LDL cholesterol (mmol L^−1^)	3.18 [2.7–3.8]	1.97 [1.6–2.54]^a^	1.9 [1.45–2.56]^a^	1.74 [1.41–2.04]^a, b, c^	<0.001
HDL cholesterol (mmol L^−1^)	1.54 [1.29–1.9]	1.33 [1.1–1.66]^a^	1.23 [1.07–1.45]^a, b^	1.15 [0.93–1.36]^a, b^	0.035
On statin (%)	14.4	90.0	72.3	89.3	<0.001
CKD STAGE N (%)					
3	8 (4.97%)	25 (13.66%)	21 (9.46%)	40 (22.60%)	<0.001
4	1 (0.62%)	0 (0%)	0 (0%)	4 (2.26%)	
5	0 (0%)	0 (0%)	0 (0%)	0 (0%)	
HbA1c (mmol mol^−1^)	39 [37–41]	40 [38–42]	57 [49–66]^a, b^	57 [50–69]^a, b^	<0.001
ACR (mg/mmol)	0.59 [0.41–0.93]	0.61 [0.4–1.14]	0.78 [0.5–1.5]^a, b^	0.93 [0.5–2.15]^a, b^	<0.001

*BMI* body mass index, *MAP* mean arterial pressure, *ABPI* ankle brachial pressure index, *CKD* chronic kidney disease, *ACR* albumin creatinine ratio

^a^Represents different from control, (p < 0.008 after Bonferonni adjustment for six intergroup comparisons)

^b^Represents different from CVD alone

^c^Represents different from diabetes alone

Individuals with T2DM were treated with diet only (19.5%), oral glucose lowering medications only (58.2%), and insulin (with or without oral glucose lowering medications, 21.0%). Due to local clinical pathways, GLP-1 based therapy was rarely used (1.3% of people with diabetes). Duration of diabetes ranged from newly diagnosed to 39.2 years (mean 9.5 years, median 8 years, IQ range 4–13). Those with CVD had a longer duration of diabetes, [mean 10.8, median 9.7 (IQ range 4.3–15) years vs. mean 8.5 median 7.7 (IQR 4–12) years; p = 0.002]. A history of myocardial infarction, CABG, PCI or acute coronary syndrome represented 56.8% of those with CVD, whilst cerebrovascular problems [ischaemic stroke or transient ischaemic attack (TIA)] represented a further 31.8%. The remaining 11.6% had a history of both cardiac and cerebrovascular disease. This distribution was the same in those with and without T2DM.

### Skin micro vascular response to acetylcholine and sodium nitroprusside

**Fig. 1:**
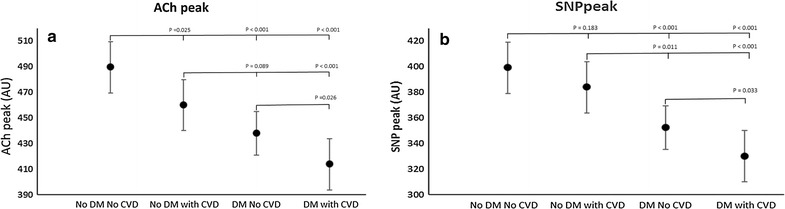
Endothelial dependent and independent function (mean ± SD) stratified by recruitment group. As multiple comparisons have been made, a p < 0.008 should be regarded as statistically significant. **a** Peak endothelial dependent responses to acetylcholine (ACh); **b** Peak endothelial independent response to sodium nitroprusside (SNP). No DM No CVD: recruited with no evidence of diabetes or overt cardiovascular disease; No DM with CVD: recruited with no evidence of diabetes, but pre-existing cardiovascular disease; DM No CVD: recruited with pre-existing diabetes but no evidence of cardiovascular disease; DM with CVD: recruited with pre-existing diabetes and cardiovascular disease

The result of the ANOVA test show that endothelium-dependent microvascular response to ACh was decreased in patients with either T2DM alone or CVD alone compared with those without either condition (Fig. 1a). There was an additive effect in patients with both T2DM and CVD, such that this group had the poorest microvascular function than either those with T2DM alone [mean ± SD 414 ± 20 vs. 438 ± 17 arbitrary units (arb.) for T2DM + CVD vs T2DM alone respectively: p = 0.026] or CVD alone (414 ± 20 vs. 460 ± 20 arb. respectively; p < 0.001), and the decline in response was numerically similar to the composite effect of each disease process separately.

The endothelium-independent response to SNP was similarly attenuated in patients with both T2DM and CVD compared with either T2DM alone (330 ± 23 vs. 352 ± 19 arb.; p = 0.03) or CVD alone (330 ± 23 vs. 384 ± 20 respectively; p < 0.001) (Fig. 1b). Again, this was numerically similar to the additive effect of either alone condition alone compared to healthy controls.

### Determinants of attenuated microvascular responses in those with DM and/or CVD

**Fig. 2:**
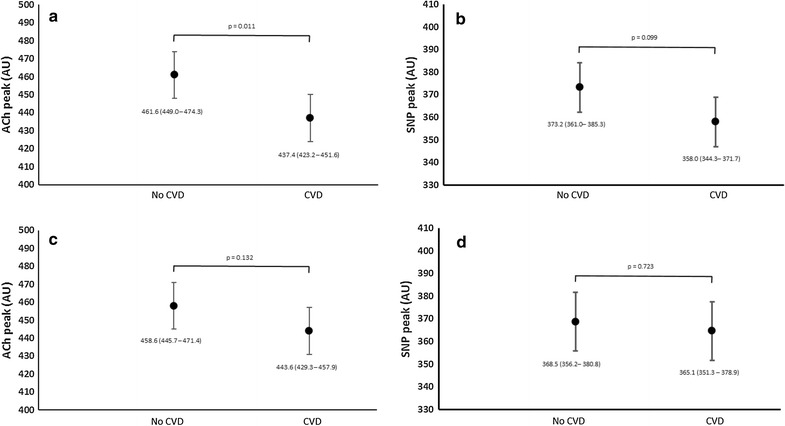
Determinants of attenuated microvascular function in those with cardiovascular disease, irrespective of diabetes, combined (mean ± 95% CI). As multiple comparisons have been made, a p < 0.008 should be regarded as statistically significant. **a** Peak endothelial dependent responses to acetyl choline (ACh) in those with and without pre-existing cardiovascular disease adjusted for age and sex (mean ± CI). **b** Peak endothelial independent response to sodium nitroprusside (SNP) in those with and without pre-existing cardiovascular disease adjusted for age and sex. **c** Peak endothelial dependent responses to acetyl choline (ACh) in those with and without pre-existing cardiovascular disease adjusted for age, sex and conventional cardiovascular risk factors (body mass index, mean arterial blood pressure, total cholesterol and smoking status). **d** Peak endothelial independent response to sodium nitroprusside (SNP) in those with and without pre-existing cardiovascular disease adjusted for age, sex and conventional cardiovascular risk factors (body mass index, mean arterial blood pressure, total cholesterol and smoking status). *CVD* participants recruited with pre-existing cardiovascular disease; *No CVD* participants recruited with no clinical history of cardiovascular disease

**Fig. 3:**
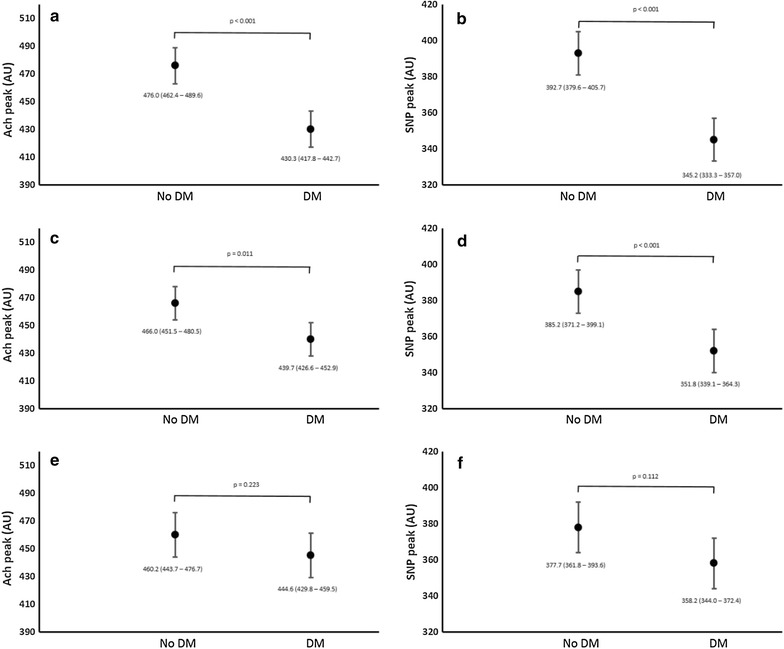
Determinants of attenuated microvascular function in those with diabetes (mean ± 95% CI), irrespective of cardiovascular disease status combined. **a** Peak endothelial dependent responses to acetylcholine (ACh) in those with and without diabetes adjusted for age and sex. **b** Peak endothelial independent response to sodium nitroprusside (SNP) in those with and without diabetes adjusted for age and sex. **c** Peak endothelial dependent responses to acetylcholine (ACh) in those with and without diabetes adjusted for age, sex and conventional cardiovascular risk factors (body mass index, mean arterial blood pressure, total cholesterol and smoking status). **d** Peak endothelial independent response to sodium nitroprusside (SNP) in those with and without diabetes adjusted for age, sex and conventional cardiovascular risk factors (body mass index, mean arterial blood pressure, total cholesterol and smoking status). **e** Peak endothelial dependent responses to acetylcholine (ACh) in those with and without diabetes adjusted for age, sex, conventional cardiovascular risk factors and HbA_1_c. **f** Peak endothelial independent response to sodium nitroprusside (SNP) in those with and without diabetes adjusted for age, sex, conventional cardiovascular risk factors and HbA_1_c. *DM* participants recruited with diabetes (including those diagnosed during screening); *No DM* participants recruited with no clinical history of diabetes and no elevated HbA1c at screening

In keeping with previous reports, in our multiple regression model conventional CVD risk factors (i.e. age, sex, BMI, serum cholesterol and mean arterial pressure) completely accounted for the effect of CVD on both microvascular endothelium-dependent (adjusted p = 0.1) and independent microvascular function (adjusted p = 0.7; Fig. 2). These risk factors could not account for the differences between those with and without T2DM (Fig. 3c, d). The addition of HbA_1_c to the model, however did account for the differences between those with and without T2DM (Fig. 3e, f).

### Exploration of the association between glycaemic control and microvascular function and the effects of duration of T2DM or comorbidities

Both endothelium-dependent and independent microvascular responses were associated with HbA_1_c after adjustment for age and sex (standardised beta, −0.154, p < 0.001; and −0.171, p < 0.001 for ACh and SNP, respectively) (Table 2). Adjustment for conventional CV risk factors did little to attenuate this association (std beta −0.093, p = <0.009 and −0.124, p < 0.001, respectively). Polypharmacy, nor any specific drug class did not impact the model, however concordant treatment algorithms across the two sites had resulted in very little heterogeneity in prescribing.

**Table 2:** Association between microvascular function and HbA1c after adjustment for duration of DM or comorbidities (CVD and CKD)

	Peak response to acetylcholine			Peak response to sodium nitroprusside	
*a: After adjustment for age and sex*					
Standardised beta	−0.160	p < 0.001	Standardised beta	−0.182	p < 0.001
*b: After adjustment for age, sex and conventional CVD risk factors (BMI, MAP, total cholesterol and smoking status)*					
Standardised beta	−0.096	p = 0.008	Standardised beta	−0.135	p < 0.001
*c: After adjustment for age, sex, conventional CVD risk factors and complex diabetes (CVD, CKD or the composite)*					
Standardised beta	−0.099	p = 0.006	Standardised beta	−0.138	p < 0.001
*d: After adjustment for age, sex, conventional CVD risk factors and duration of diabetes*					
Standardised beta	−0.043	p = 0.3	Standardised beta	−0.105	p = 0.02

Standardised beta regression coefficient between microvascular function and HbA1c after adjustment for potential confounders and putative modifiers. The presence of CVD or CKD were given equal weight, the combination of both was doubly weighted

*DM* type 2 diabetes mellitus, *CVD* clinically confirmed cardiovascular disease, *CKD* chronic kidney disease (defined as an eGFR ≤60 mL/min)

#### Comorbidities

Inclusion of CVD, CKD or the composite of the two made no impact on the association between HbA_1_c and microvascular function, either numerically or statistically (adjusted std beta −0.094; p = 0.008 and −0.125; p < 0.001, for endothelial dependent and independent perfusion respectively).

#### Duration of diabetes

Adjustment for the duration of diabetes entirely accounted for the association between HbA_1_c and endothelium-dependent microvascular function (adjusted std beta −0.041; p = 0.3). Endothelium-independent function, however, maintained its association with HbA_1_c, albeit attenuated to borderline significance after adjustment for duration of diabetes (adjusted std beta −0.092; p < 0.05). The inclusion of other cardiovascular risk factors or comorbidities did not alter this association.

### Endothelial function as measured by ENDOPAT

The RHI, measuring endothelial response to occlusion, was significantly different between the four groups (ANOVA p < 0.001: Additional file 1: Figure S2). After adjustment for age, sex and the presence of diabetes, CVD showed a trend towards reducing RHI in this subgroup analysis (2.47 ± 0.72 for those without CVD vs 2.31 ± 0.75 for those with CVD; p = 0.02). Diabetes significantly attenuated RHI in the population as a whole (p < 0.001) and when stratified into those with and without CVD, such that in those without CVD, the presence of diabetes reduced RHI from 2.71 ± 0.65 to 2.32 ± 0.68 (p < 0.001) and in those with pre-existing CVD a similar decline was observed (2.52 ± 0.68 vs 2.21 ± 0.62 respectively; p = 0.005).

### Exploration of the association between glycaemic control and ENDOPAT and the effects of duration of T2DM or comorbidities

RHI was associated with HbA_1_c after adjustment for age and sex (standardised beta, −0.172, p < 0.001). Inclusion of CVD, CKD or the composite of the two made no impact on the association between HbA_1_c and RHI, either numerically or statistically (adjusted std beta −0.171 p < 0.001). In an analysis reflecting the results of the microvascular response to ACh, adjustment for the duration of diabetes entirely accounted for the association between ENDOPAT measured endothelial function and glycaemic control (adjusted std beta −0.009; p = 0.902)

## Discussion

The results of this study demonstrate for the first time that the microvascular dysfunction associated with T2DM and CVD are additive. This suggests different mechanisms of deterioration in microvascular dysfunction for the two disease processes. Supporting this finding, we have shown that the differences between those with and without CVD are accounted for by traditional CVD risk factors, whereas the addition of glycaemic control to the model is required to account for the microvascular dysfunction due to the presence of T2DM. Further, we have demonstrated that this model is essentially unaffected by the complexity of disease suggesting links between glycaemic control and microvascular function are also relevant in complicated diabetes. These findings were mirrored in the only alternative FDA-approved measure of endothelial function, suggesting our results represent a systemic disposition, rather than localised to the skin alone.

If our findings were prospectively validated, one could argue that guidelines advocating relaxation of targets for glycaemic control in those with multi-morbidity are potentially missing an opportunity to slow progression of microvascular disease, assuming the targets can be achieved without adverse metabolic consequences such as hypoglycaemia and weight gain. Duration of T2DM, however, accounts for the association between glycaemic control and endothelium-dependent microvascular function. Again, if prospectively reproduced, this would have important implications for setting of targets for glycaemic control in those with advanced T2DM. Whilst our data may suggest that occurrence of CVD and/or CKD alone should not cause a relaxing of targets for glycaemic control, the benefit from tight glycaemic control on the microcirculation may be lost in those with prolonged duration of disease. It is important to stress, however, that these findings would need to be validated in prospective randomised control trials, individualising treatment targets based on patients’ characteristics. To date, however, only one study has even attempted to demonstrate the feasibility of individualising care [23]. Whilst the INTERVAL study demonstrated that achieving individualised glycaemic targets was feasible, process of setting such targets themselves was very difficult [24].

It is well established that CVD and T2DM independently affect microvascular function [25–27]. Previous studies assessing microvascular response to ACh and SNP in well-established DM with concomitant acute and chronic CVD, however, where inconclusive due to small number of patients [11–14]. As a result, they were unable to tease out the independent effects of large vessel disease in those with CVD from the adverse glycaemic legacy associated with a long duration of diabetes.

Although microvascular dysfunction in those with CVD is explained by cardiovascular risk factors [25], the mechanism through which sustained hyperglycaemia causes vascular complications is less clear. Contemporaneous optimal glycaemic control is not associated with improved coronary microvascular response, suggesting that the adverse function may be as a result of glycaemic legacy effect [28]. It is generally understood that hyperglycaemia acts on endothelial cell function [29] and resultant microvascular endothelial dysfunction perpetuates a cycle with a reduction in insulin-mediated glucose uptake. Further, the long-term protection granted by early good glycaemic control in diabetes (legacy effect) remains unclear. The idea of a legacy effect of tight glycaemic control was first suggested by the results of UKPDS trial [4]. The exact mechanisms of glycaemic legacy are not known but epigenetic changes seem to be involved [30]. In fact, hyperglycaemia influences gene expression both directly, inducing histone acetylation and methylation, and indirectly through downstream intracellular pathways triggered by oxidative stress which also lead to AGE (advanced glycation end-products) formation [29, 31].

Clinicians are often faced with difficulties when attempting to interpret recent guidelines for the management of hyperglycaemia in T2DM, which advocate less stringent glycaemic target for a spectrum of patient factors including motivation, support networks, duration of disease and complex diabetes [8]. These chosen parameters, however, are based on consensus rather than clear evidence. Our work, by demonstrating that the association between microvascular function and glycaemic control is accounted for by duration of diabetes but not complexity of disease, suggests that the current guidance requires some prospective validation. Further, it also questions guidance to relax glycaemic targets in those with newly diagnosed disease but significant co-morbidities. The findings clearly need to be verified in longer term studies, or by meta-analyses of existing data from the landmark clinical trials. ACCORD, VADT and ADVANCE-ON [5–7] demonstrated some benefit of tight glycaemic control in slowing the microvascular complications in patients with advanced/complex T2DM, although no reduction in cardiovascular events or mortality. Further analyses of these data may elucidate whether there is a dominant effect of prolonged adverse glycaemic legacy or concomitant large vessel disease, on microvascular outcomes.

### Study limitations

This work has several limitations, most pertinently, the cross-sectional nature of our study prevents any causal or temporal relationships, as well as any physiopathological inferences. Further, it limits the possibility to address the question of a threshold effect for duration of T2DM and association between HbA_1_c and microvascular function. More studies of a longitudinal nature are needed to answer such questions. Secondly, although geographically distinct, both centres studied a largely white Caucasian population so not necessarily translatable to other ethnic groups who have a high incidence of T2DM. Finally, the use of skin as a marker of systemic microvascular dysfunction has its critics. Previous studies, however, have shown that the skin microvascular bed can be used as a valid marker of more generalised microvascular function [17, 32]. Further support for this is given in a recent systematic review and meta-analysis which demonstrated that several different markers of microvascular function, including skin (ACh-mediated responses), eye, and kidney, are associated with type 2 DM, suggestive of common underlying pathways in different tissues [33]. The project is strengthened by the multi-centre design, size of this population and the detailed characterisation of the participants.

## Conclusion

In conclusion, our results indicate that microvascular dysfunction is different in CVD and T2DM and therefore should be regarded independently. This is important new information because microvascular complications, such as retinopathy and nephropathy, have the greatest impact on a patient’s quality of life. In order to slow or halt progression of microvascular complications in those with T2DM, glycaemic control should be aggressively targeted in early T2DM, even if CVD already exists. Current guidelines advocate relaxing glycaemic targets in prolonged T2DM, even if the patient is otherwise healthy. Our findings are in keeping with these recommendations, as the association between microvascular function and glycaemia appears to be lost, however still require prospective validation.

## Additional file

**Additional file 1: Figure S1.** Schematic representations of the protocols used to deliver Acetylcholine (ACh) and Sodium Nitroprusside (SNP). Each 20 s scan is represented by a section on the horizontal line, section with an arrow represent scan during which the drugs were delivered. **Figure S2.** Reactive Hyperaemic Index (RHI) performed in a subgroup of the total cohort stratified by recruitment group. As multiple comparisons have been made, a p < 0.008 should be regarded as statistically significant. No DM No CVD: (n = 107) Recruited with no evidence of diabetes or overt cardiovascular disease; No DM with CVD: (n = 129) Recruited with no evidence of diabetes, but pre-existing cardiovascular disease; DM No CVD: (n = 117) Recruited with pre-existing diabetes but no evidence of cardiovascular disease; DM with CVD: (n = 105) Recruited with pre-existing diabetes and cardiovascular disease.
