# Supplementary material for: The impact of cardiovascular co-morbidities and duration of diabetes on the association between microvascular function and glycaemic control
Source: Cardiovasc Diabetol. 2017 Sep 15;16:114. doi: 10.1186/s12933-017-0594-7 (PMC5603035; doi:10.1186/s12933-017-0594-7)
Supplement: Supplementary file 1 — Additional file 1: Figure S1. Schematic representations of the protocols used to deliver Acetylcholine (ACh) and Sodium Nitroprusside (SNP). Each 20 s scan is represented by a section on the horizontal line, section with an arrow represent scan during which the drugs were delivered. Figure S2. Reactive Hyperaemic Index (RHI) performed in a subgroup of the total cohort stratified by recruitment group. As multiple comparisons have been made, a p < 0.008 should be regarded as statistically significant. No DM No CVD: (n = 107) Recruited with no evidence of diabetes or overt cardiovascular disease; No DM with CVD: (n = 129) Recruited with no evidence of diabetes, but pre-existing cardiovascular disease; DM No CVD: (n = 117) Recruited with pre-existing diabetes but no evidence of cardiovascular disease; DM with CVD: (n = 105) Recruited with pre-existing diabetes and cardiovascular disease. [file 12933_2017_594_MOESM1_ESM.docx]

ADDITIONAL FIGURES

**Figure S1. Schematic representations of the protocols used to deliver Acetyl Choline (Ach) and Sodium Nitroprusside (SNP).**


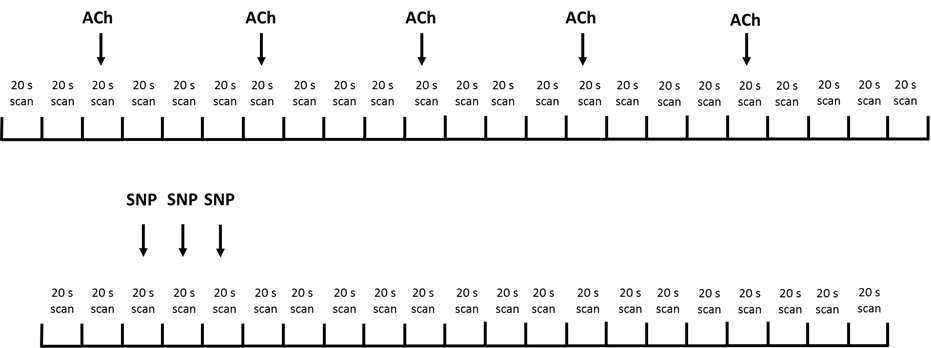


**Figure S2. RHI stratified by recruitment group.**
